# Behavioral and Neuropathological Changes After *Toxoplasma gondii* Ocular Conjunctival Infection in BALB/c Mice

**DOI:** 10.3389/fcimb.2022.812152

**Published:** 2022-03-09

**Authors:** Gabrielly Lisboa da Silva Soares, Ellen Rose Leandro Ponce de Leão, Sinara Franco Freitas, Raissa Maria Carvalho Alves, Naiana de Paula Tavares, Maria Vitória Nascimento Costa, Gabriel Castro de Menezes, Jhonnathan Henrique Palheta de Oliveira, Luma Cristina Ferreira Guerreiro, Alexa Camila Lopes de Assis, Sanderson Corrêa Araújo, Felipe Tuji de Castro Franco, Ana Karyssa Mendes Anaissi, Ediclei Lima do Carmo, Rafaela dos Anjos Pinheiro Bogoevich Morais, Samia Demachki, José Antonio Picanço Diniz, Heloisa Marceliano Nunes, Daniel C. Anthony, Daniel Guerreiro Diniz, Cristovam Wanderley Picanço Diniz

**Affiliations:** ^1^ Laboratório de Investigações em Neurodegeneração e Infecção, Instituto de Ciências Biológicas, Hospital Universitário João de Barros Barreto, Universidade Federal do Pará, Belém, Pará, Brazil; ^2^ Laboratório de Microscopia Eletrônica, Instituto Evandro Chagas, Belém, Pará, Brazil; ^3^ Laboratório de Anatomia Patológica, Hospital Universitário João de Barros Barreto, Universidade Federal do Pará, Belém, Pará, Brazil; ^4^ Laboratório de Toxoplasmose, Instituto Evandro Chagas, Ananindeua, Pará, Brazil; ^5^ Seção de Hepatologia, Instituto Evandro Chagas, Belém, Pará, Brazil; ^6^ Department of Pharmacology, Laboratory of Experimental Neuropathology, University of Oxford, Oxford, England, United Kingdom

**Keywords:** *Toxoplasma gondii*, ocular conjunctival instillation, neuroinfection, hippocampus, behavioral tests, microglia response

## Abstract

Ocular infection with *Toxoplasma gondii* causes toxoplasmosis in mice. However, following ocular infection with tachyzoites, the cause of the accompanying progressive changes in hippocampal-dependent tasks, and their relationship with the morphology and number of microglia, is less well understood. Here, in 6-month-old, female BALB/c mice, 5 μl of a suspension containing 48.5 × 10^6^ tachyzoites/ml was introduced into the conjunctival sac; control received an equal volume of saline. Before and after instillation, all mice were subject to an olfactory discrimination (OD) test, using predator (cat) feces, and to an open-field (OF) task. After the behavioral tests, the animals were culled at either 22 or 44 days post-instillation (dpi), and the brains and retinas were dissected and processed for immunohistochemistry. The total number of Iba-1-immunolabeled microglia in the molecular layer of the dentate gyrus was estimated, and three-dimensional reconstructions of the cells were evaluated. Immobility was increased in the infected group at 12, 22, and 43 dpi, but the greatest immobility was observed at 22 dpi and was associated with reduced line crossing in the OF and distance traveled. In the OD test, infected animals spent more time in the compartment with feline fecal material at 14 and at 43 dpi. No OD changes were observed in the control group. The number of microglia was increased at 22 dpi but returned to control levels by 44 dpi. These changes were associated with the differentiation of *T. gondii* tachyzoites into bradyzoite-enclosed cysts within the brain and retina. Thus, infection of mice with *T. gondii* alters exploratory behavior, gives rise to a loss in predator’s odor avoidance from 2 weeks after infection, increased microglia number, and altered their morphology in the molecular layer of the dentate gyrus.

## Introduction

*Toxoplasma gondii* is an intracellular parasite that can infect most warm-blooded animals, where it may invade the central nervous system and provoke neuroinflammation and behavioral changes (for recent reviews, see Mendez and Koshy, 2017; Martinez et al., 2018; Chen et al., 2019; de Haan et al., 2021; Nasuhidehnavi and Yap, 2021; Nayeri et al., 2021; Postolache et al., 2021). It is estimated that approximately 30% of the world’s population is infected with this protozoan, but there is much greater prevalence in African and South American countries (Agordzo et al., 2019; Karshima and Karshima, 2020; Rahmanian et al., 2020; de Lima Bessa et al., 2021). For example, in Brazil seroprevalence is 92%, but in the USA, it is only 22.5% (Jones et al., 2001; Jones and Dubey, 2014).

Acute *T. gondii* infection is associated with the rapid replication of tachyzoites followed by their conversion to bradyzoites, which can form infectious cysts within the brain, muscle, and other tissues, which establishes a life-long, latent infection (Sullivan and Jeffers, 2012; Rougier et al., 2017) that can give rise to neuroinflammation, vascular injury, and damage to the blood–brain barrier (Castaño Barrios et al., 2021; Egorov et al., 2021).

It has been suggested that the ability of *T. gondii* to alter the behavior of its intermediate hosts may enhance its transmission rate through the food chain. In rodents, for example, motor impairments, longer reaction times, and a reduction in the avoidance of feline predators, as well as spatial learning and memory impairments, have all been reported to be features of a *T. gondii* infection (Webster, 2001; Hodkova et al., 2007; Webster, 2007; Flegr, 2010; Berenreiterová et al., 2011; Flegr et al., 2011; Afonso et al., 2012; Flegr and Markoš, 2014; Mahmoudvand et al., 2015; Torres et al., 2018). However, the effect of *T. gondii* infection on the behavior of mice in the open field is not straightforward. Some report no changes in the locomotor activity (Evangelista et al., 2021), others describe reduced activity (Mahmoudvand et al., 2015; Bezerra et al., 2019), and others report hyperactivity (Meurer et al., 2020) in infected mice. Similarly, it has been reported that infection can either increase or decrease anxiety-like behaviors (Mahmoudvand et al., 2015; Torres et al., 2018; Boillat et al., 2020), and the selective loss of innate specific feline-evoked avoidance in *T. gondii*-infected mice also remains a controversial issue (Worth et al., 2013; Worth et al., 2014; Boillat et al., 2020). However, consistent losses in the aversion of felid urine or feces have been described in infected rats and mice (Berdoy et al., 2000; Vyas et al., 2007; Lamberton et al., 2008; Kannan et al., 2010; Ingram et al., 2013; Vyas, 2015; Hammoudi and Soldati-Favre, 2017). Boillat and colleagues demonstrated that *T. gondii* lowers general anxiety in infected mice and that the lack of aversion of mice infected with *T. gondii* extends to predators other than felids (Boillat et al., 2020). They have also shown that cyst load in the host is associated with transcriptional changes in markers of inflammation (Boillat et al., 2020). Other studies suggest that the behavioral changes might be associated with impaired long-term fear memory consolidation through dysfunctional cortical and amygdaloid circuits of infected mice (Ihara et al., 2016). Indeed, following *T. gondii* infections, the molecular layer of the dentate gyrus (MolDG) may be a specific target (van Groen et al., 2003) leading to memory dysfunction (Berenreiterová et al., 2011).

Studies in humans infected with *T. gondii* have shown an olfactory preference for the odor of domestic cat urine compared to larger cats (Flegr et al., 2011). By analyzing the response to the urine of different animals, they found an increase in the attractiveness of cat urine odor in human males but not in females. They suggested that an important factor in the species’ territorial demarcation process, the amino acid felinine (Hendriks et al., 1995), which is secreted in the urine of small cats (feline subfamily), might be involved in the altered attraction in humans.

Previous findings in BALB/c and C57BL/6 mouse models, using transcriptomic analysis in chronic *T. gondii* infection, have revealed that there are significant changes in host and parasite gene expression as the disease progresses (Bergersen et al., 2021) and in the host metabolome. For example, studies in BALB/c mice have shown that *T. gondii* induces significant metabolic disturbances in the hippocampus and cerebellum at 7, 14, and 21 days postinfection, many of which were described as potential biomarkers for *T. gondii* infection (Ma et al., 2020; Ma et al., 2021). Another study has also revealed changes in the cortex, where there is a progressive increase in unsaturated fatty acid biosynthesis to promote growth and survival of the protozoan (Ma et al., 2019). Finally, it has been highlighted that there is an important role for the vomeronasal organ, the Grueneberg ganglion, and the chemosensory neurons within the main olfactory epithelium, in which nerve endings are exposed to kairomones, which are predator-derived chemostimuli (Pérez-Gómez et al., 2015). These authors described a highly organized parallel subsystems from the periphery converging to specific subregions of the ventral amygdala and the ventromedial hypothalamus that process the signatures of volatile and non-volatile kairomones through the activation of multiple olfactory subsystems.

While controversial, it has been suggested that *T. gondii* exhibits significant tropism for immune-privileged tissues such as the eyes, brain, and testes (Schlüter and Barragan, 2019). Indeed, *T. gondii* can infect virtually any nucleated cell within and outside the CNS and the host’s immune response is critical for limiting the acute phase of infection, but also for promoting encystment during repeated cycles of clinical exacerbation, which is essential for keeping individuals chronically infected (Tong et al., 2021). Microglial cells represent the most important component of the primary innate immune response of the central nervous system (Perry, 2016; Garaschuk and Verkhratsky, 2019; Sierra et al., 2019), and our preliminary data in BALB/c mice following the instillation of *T. gondii* tachyzoites into the ocular conjunctival sac suggest a possible association between behavioral abnormalities and microglial behavior (Normando et al., 2017).

Using *T. gondii* as the conjunctival sac model in BALB/c mice the present study examined tachyzoite differentiation bewteen bradyzoites in different CNS compartments and the microglial changes in the MolDG using a stereological approach (West, 2002).

## Methods

Female BALB/c mice were maintained in animal housing in accordance with the guidelines published by the National Institutes of Health (Guide for the Care and Use of Laboratory Animals). The experimental protocol was submitted and approved prior to study initiation by the Ethics Committee on Experimental Animal Research (from the Institute of Biological Sciences, Federal University of Pará, Brazil, CEUA no. 7961160818) *and from the Ethics Committee of Evandro Chagas Institute (Protocol No. 09/2021)*. The procedures, involving the handling of the parasites, were carried out in compliance with the preestablished standards and criteria required by the International Biosafety Committee. In this study, thirty BALB/c isogenic adult female mice, at 4 months of age, were obtained from the animal services unit of Instituto Evandro Chagas. The animals were housed in standard laboratory cages, with ad libitum access to water and food and were kept in a temperature-controlled room (23 ± 2°C) under a 12-h light–dark cycle. Tachyzoites of *T. gondii* RH strain (Genotype I) were obtained from the Toxoplasmosis Laboratory of Instituto Evandro Chagas.

**Figure 1:**
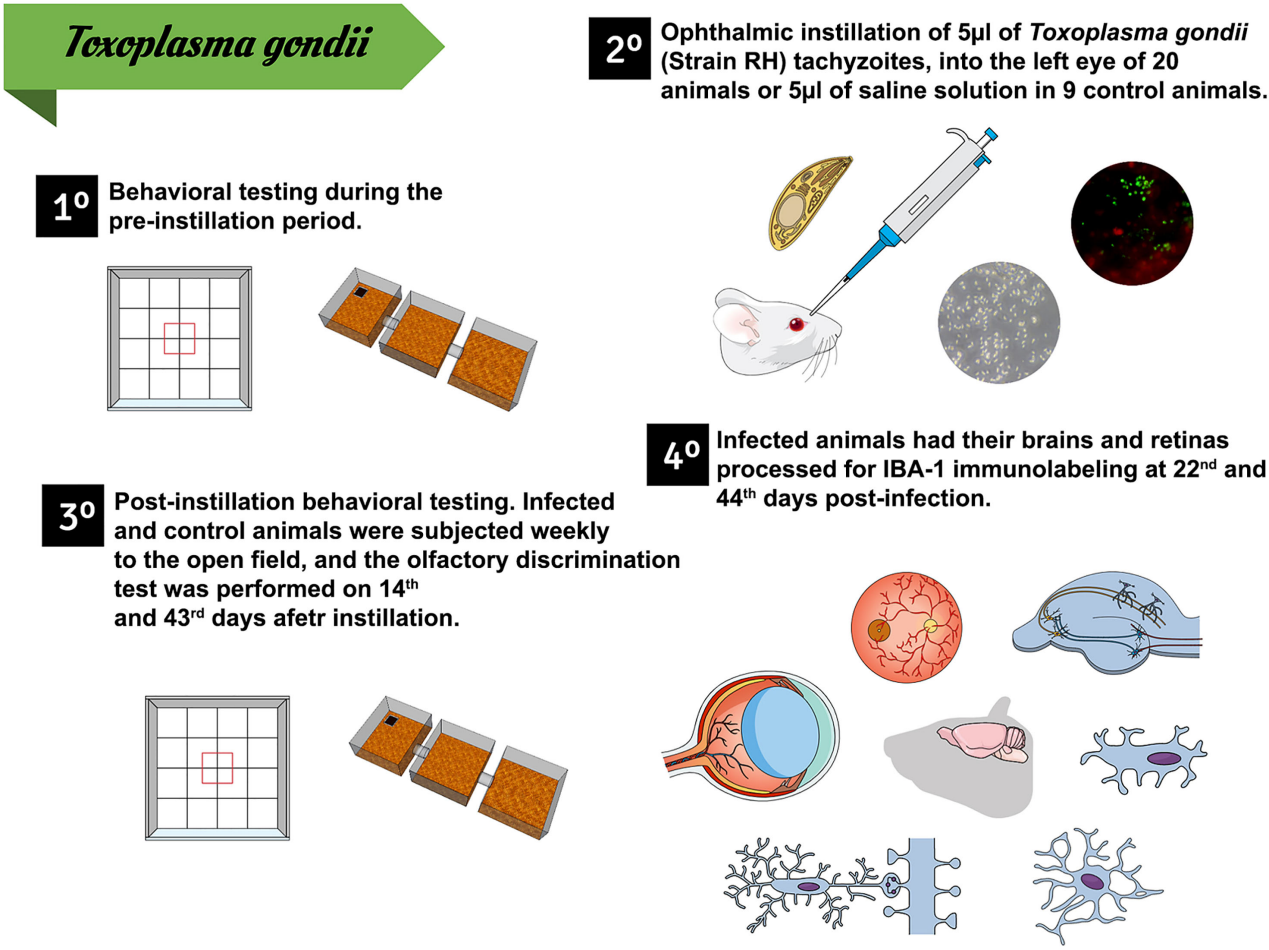
Experimental timeline. 1. Days 1 to 4: behavioral tests performed before conjunctival instillation. 2. Ophthalmic instillation of either an infected suspension (n = 20) or equal volume of saline (n = 9), 65 days after starting the behavioral tests. 3. Behavioral tests at 22 and 44 dpi. 4. Animal euthanasia and histological procedures at 22 and 44 dpi.

The animals were anesthetized with avertin (0.08 mg/5 g of body weight) and had the conjunctival sac of the left eye instilled either with 5 μl of an infected suspension containing 48.5 × 10^6^ parasites/ml (infected group) or with an equal volume of saline solution (control group). Five infected and five control subjects were sacrificed at 22 dpi and the other five individuals from each group were sacrificed at 44 dpi. **Figure 1** shows the experimental timeline.

Before and after ophthalmic instillation, all subjects were submitted to open-field (OF) and olfactory discrimination (OD) tests.

### Open-Field Test

Open arenas are widely used for measuring anxiety-like behavior in mice and rats. In these tasks, when animals explore an unfamiliar area, they remain close to the walls; this preference is taken as an indication of fear-induced anxiety (Lalonde and Strazielle, 2008; Ennaceur, 2014), and such “hiding” behavior may reduce attack and predation, and it is included in the repertoire of animal survival instincts (Ennaceur, 2014).

The OF apparatus consisted of a gray polyvinyl chloride box (30 cm × 30 cm × 40 cm), with a floor digitally divided into central and peripheral regions of equal areas; see **Supplementary Figure 1A**. For the OF test, the animals were placed individually in the center of the arena and their exploratory activity was monitored for 3 min. All experiments were carried out following the same protocol at different timepoints. The test was recorded by a video camera placed 1 m above the apparatus. We measured distance traveled, immobility, lines crossed, and the contrast between time spent in the periphery and in the center (C = (Tp – Tc)/(Tp + Tc), where C = contrast, Tp = time spent in the periphery, Tc = time spent in the center. The apparatus was sanitized with 70% alcohol at the end of each test to remove any remaining odors. The OF test was performed weekly in the infected and control groups to assess behavioral changes as the disease progressed.

### Olfactory Discrimination Test

An OD test was performed in a box divided into 3 compartments following the previous protocol (Soffié and Lamberty, 1988). The compartments were placed side by side and connected with polyvinyl tubes allowing mice to move between compartments (see **Supplementary Figure S1B**). All animals were adapted to the apparatus, exploring clean compartments (no straw), for 5 min. After adaptation, alcohol 70% was used to clean the apparatus. To perform the olfactory test, the central compartment contained clean straw, and each of the lateral compartments contained spoiled straw originating from a cage of mice undergoing testing and clean straw plus feline feces, respectively. The animals were placed, one at a time, facing the wall, in the central compartment with the clean straw, and a camera recorded their behavior for 3 min. The amount of time spent in the compartment with cats’ feces in relation to the total time of the OD test was estimated as follows: C = (Tt – Tf)/(Tt + Tf), where C = contrast, Tt = total time, and Tf = time in the compartment with cats’ feces. Because Tt is a fixed value (180 s) and Tf varies as a function of mouse preferences, as C values decrease, time spent in the compartment with cats’ feces increases. The contrast index was used to normalize data to each mouse’s performance, thus accounting for the variation in performance between individuals (Torres et al., 2006; Diniz et al., 2010).

### Histological and Immunohistochemical Procedures

When each animal reached the designated survival time, final behavioral tests were performed, and then the mice were weighed and anesthetized with intraperitoneal 2,2,2-tribromoethanol (0.15 ml/g of body weight) and transcardially perfused with heparinized saline, followed by 4% paraformaldehyde in 0.1 M phosphate buffer (pH 7.2–7.4). Anatomical serial sections (80 µm thickness) were obtained using a vibratome (Microm, model HM 650 MK, Microm International GmbH, Walldorf, Germany) and immunolabeled using polyclonal antibodies. Brain sections were pretreated in 0.2 M boric acid (pH 8.0) at 70°C for 60 min for antigen retrieval. Afterward, they were washed in a 0.1-M saline Tris buffer solution, pH 7.2–7.4, immersed for 60 min in 10% casein, and then incubated in a primary antibody (Anti-IBA-1 polyclonal antibody, Rabbit/Wako, code 01127991, Wako Pure Chemical Industries Ltd., Osaka, Japan) diluted in Tris buffer saline (pH 7.0), 1:500, for 72 h. Selective immunolabeling for *T. gondii* antigens was achieved with polyclonal antibodies produced by the Laboratory of Toxoplasmosis at the Instituto Evandro Chagas, as follows: BALB/c mice were orally inoculated with 25 µl of a suspension containing 10 tissue cysts of *T. gondii* cystogenic strains (VEG) diluted in 0.9% saline. After 40–45 days of inoculation, blood aliquots of 0.3 ml were collected by submandibular puncture. Blood serum was separated by centrifugation (2,500 g × 10 min) and kept at -20°C until the serological test was performed. For detection of total antibodies anti-*T. gondii*, the modified agglutination test was used (Dubey & Desmonts, 1987). Aliquots of serum were diluted from 1:10 to 1:1,024 and placed to react with antigen from the RH strain in microtiter plates. Positive serum dilutions were identified by homogeneous mesh covering at least 50% of the well cavity. The cutoff dilution was 1:10. Negative samples were identified by antigen precipitation as a ring at the bottom of the well. Positive samples were adsorbed in brain homogenate/PBS (1:10 V/V), in the proportion of (1:1 V/V), refrigerated for 12 h at 10°C. Afterward, this material was centrifuged at 3,000 rpm, and the supernatant was collected for later use.

Free-floating retinas and brain sections were subject to the same *T. gondii* antigen detection using adaptation procedures. Brain sections were submitted to antigen recovery in 0.2 M boric acid solution pH 9.0 at 70°C for 1 h. These sections were then permeabilized with detergent (5% Triton X-100 in 0.1 M saline phosphate buffer) for 5 min and then incubated in a 10% casein solution in 0.1 M saline phosphate buffer (Vector Laboratories, Burlingame, CA) to reduce non-specific labeling.

After washing, all retinas were immersed for 10 min in collagenase 0.01% in 0.1 M PBS (pH 7.2–7.4) at room temperature to increase tissue penetration of antibodies. Retinas were then washed in PBS and incubated for 15 min in 10% methanol + 3% hydrogen peroxide in 0.1 M PBS followed by a 5-min immersion in 0.1 M PBS Triton 5% twice.

After three washes in 0.1 M phosphate buffer, 5 min each, brain sections and retinas were subjected to a Mouse-on-Mouse (MOM) protocol (M.O.M. kit, Vector Laboratories, Burlingame, CA, USA) as follows: MOM IgG blocking for 1 h, primary antibody for 72 h (anti-*T. gondii* 1:10, provided by Instituto Evandro Chagas, PA, Brazil, or anti-Iba1, 2 µg/ml, #019-19741; Wako Pure Chemical Industries, Ltd., Osaka, Japan) diluted in 0.1 M PBS (pH 7.2–7.4). Brain sections and free-floating retina were then washed in 0.1 M PBS and incubated overnight in a solution containing a secondary antibody (biotinylated, Anti-Mouse IgG, Anti-Rabbit IgG, Vector, code ZB0924, BA-1,400). Sections and retina were then submitted to deactivation of endogenous peroxidase, by immersion in 0.3% hydrogen peroxide (H_2_O_2_), washed in PBST, and then immersed in a solution of the avidin–biotin complex (VECTASTAIN ABC kit; Vector Laboratories^®^) for 60 min. Sections were washed and reacted to visualize horseradish peroxidase (HRP) enhanced by the glucose oxidase–DAB–nickel method (Shu et al., 1988). This procedure improved the background and foreground contrast. To submit brain sections and retinas to the glucose oxidase–DAB–nickel method, all sections, and retina were washed again in PBS before incubation in 0.2 M acetate buffer (pH 6.0) for 5 min and revealed in GND solution (diaminobenzidine 0.6 mg/ml, ammonium nickel chloride 2.5 mg/ml, and glucose oxidase 1 mg). All steps were carried out under gentle and constant agitation. Finally, the retinas were mounted between two glass slides, one of which was gelatinized to provide adherence, and sandwiched retinas remained between glass slides overnight. Brain sections were mounted in gelatinized slides and dried at room temperature. After drying, all retinas and brains sections were counterstained by Giemsa and cresyl violet respectively and then submitted to alcohol and xylene series for dehydration and clearing and then coverslipped with embedding medium (Entellan, Merck Millipore, Darmstadt, Germany).

We evaluated the specificity of the immunohistochemical patterns by omitting the primary antibody (Saper and Sawchenko, 2003) that revealed no unspecific labeling.

### Stereological Counting Procedures

After microglial selective immunolabeling, we estimated the numbers of IBA-1-immunolabeled cells in both control and infected mice. We used the optical fractionator to determine cell numbers (West, 1999; West, 2002; Bonthius et al., 2004). The optical fractionator is unaffected by histological changes, shrinkage, or damage-induced expansion of tissue (West et al., 1991). At all levels in the histological sections, we delineated the Mol-DG layer, digitizing directly from sections using a low-power ×4 objective. The microscope and motorized stage system were coupled to a computer-running Stereo Investigator and Neurolucida software (MicroBrightField, Williston, VT) used to store and analyze x, y, and z coordinates of the digitized points. High-power images were acquired under oil immersion, with a high-resolution, ×100 oil immersion plan fluoride objective (Nikon, NA 1.3, DF = 0.19 µm), and a computer-running Stereo Investigator software (MBF Bioscience Inc., Frederick, MD, USA) which was used to store and analyze the x, y, and z coordinates of the digitized points. In each counting box, the section thickness was carefully assessed using the high-power objective, defining the top and bottom of the section. All microglial cell bodies that came into focus within the counting box were counted and added to the total sample of markers. This required them to be entirely contained in the counting box or to cross acceptance lines without touching rejection lines (Gundersen and Jensen, 1987).

**Supplementary Figure 2** shows a low-power picture of the molecular layer of the dentate gyrus (shaded area—A) and grid used to count cells with counting boxes displaying acceptance (green) lines and rejection (red) lines (B).

Because cell thickness and cell distribution were uneven at each counting site, the total cell number estimate was based on the number-weighted section thickness. The counting boxes were systematically and randomly distributed within a grid, the dimensions of which were chosen to achieve an acceptable methodological error coefficient (CE <0.05). We have chosen the previously tested and validated coefficient of Scheaffer which seems to be closer to the true error (Glaser and Wilson, 1998). The ratio between the intrinsic methodological error (Scheaffer coefficient) and the coefficient of variation defines the acceptable error level of the stereological estimates (Slomianka and West, 2005). The CE expresses the accuracy of the cell number estimates. The experimental parameters for grid and counting boxes for microglia were established in pilot experiments and then uniformly applied to all animals.

To estimate the total number of cells, the optical fractionator multiplies the total number of markers counted within each counting box by three sampling fractions, representing the number of counted sections relative to the total number of sections, the area of the counting box relative to the grid area, and the thickness of the counting box in relation to the section thickness after histological procedures. These fractions designated section sampling fraction or ssf, area sampling fraction or asf, and thickness sampling fraction or tsf when multiplied by the total markers originate the equation that estimates the total number of cells as follows:


N=ΣQ∗1/ssf∗1/asf∗1/tsf

where N is the total number of cells and ΣQ is the number of counted objects (markers).

In this study, the number of sections sampled was 1/4, the height of the dissector was 15 µm, and the dimensions of the counting box and the grid (x and y steps), were 50 × 50 µm and 70 × 70 µm, respectively. **Table 1** shows detailed stereological parameters.

**Table 1:** Microglial stereological estimate in the molecular layer of dentate gyrus.

Subjects	Microglia total number	Thickness (µm)	Coefficient of error (Scheaffer)
		**Control**
**09**	4.512	43.4	0.056
**08**	5.932	38.3	0.046
**01**	3.297	24.3	0.049
**10**	3.656	27.3	0.048
**Mean**	4.349	33.3	0.049
**SD**	1.171	9.0	0.043
**CV**	0.26		
**CV²**	0.067		
**CE^2^**	0.002		
**CE²/ CV²**	0.29		
**CV² - CE²**	0.065		
**CVB²(%)**	97.01		
		**22 days postinfection**
**22**	6.542	29.7	0.037
**26**	6.005	24.9	0.040
**28**	5.062	25.5	0.037
**15**	5.914	28.6	0.041
**18**	5.828	33.6	0.040
**Mean**	5.870	28.46	0.039
**SD**	530.6		
**CV**	0.09		
**CV²**	0.008		
**CE^2^**	0.001		
**CE²/ CV²**	0.125		
**CV² - CE²**	0.007		
**CVB²(%)**	87.5		
		**44 days postinfection**
**27**	5.713	29.0	0.037
**24**	6.629	36.6	0.039
**11**	4.525	25.6	0.041
**16**	5.346	30.6	0.038
**29**	5.828	33.8	0.040
**Mean**	5.608	31.12	0.039
**DP**	765.4		
**CV**	0.136		
**CV²**	0.018		
**CE^2^**	0.001		
**CE²/CV²**	0.055		
**CV² - CE²**	0.017		
**CVB² (%)**	94.4		

Results from control and infected mice 22 days postinfection and 44 days postinfection.

CE, coefficient of error (Scheaffer); CVB^2^, coefficient of biological variation; SD, standard deviation; CV, coefficient of variation. CVB^2^ (%) = CVB^2^ × 100/CV^2^.

**Table 1**. Microglial stereological estimate in the molecular layer of dentate gyrus. Results from control, and infected mice 22 days post-infection and 44 days post infection.

Stereological results from different experimental groups were compared using parametric statistical analyses with two-tailed t-tests. Differences between groups were accepted as significant at a 95% confidence level (p < 0.05).

### Three-Dimensional Reconstructions

Microglia from molecular DG were analyzed under oil immersion using a ×100 oil immersion, high-resolution, plan fluoride objective (Nikon, numeric aperture—NA 1.3, depth of field—DF = 0.19 µm).

Images for 3D reconstructions were acquired, and the morphological features of microglia were digitized point by point with Neurolucida software (MBF Bioscience Inc., Frederick, MD). We performed 3D reconstructions only on cells with unequivocally complete arbors, discarding cells with branches that were artificially cut (at the surface or the bottom of sections) or not fully immunolabeled. Terminal branches were typically thinner. Microglia were selectively immunolabeled with IBA-1 antibody and photomicrography’s correspondence to different magnifications of microglia from 6-month-old mouse Mol-DG.

### Statistical Analysis

Statistical analysis of ANY-maze findings was done with BioEstat 5.4 and GraphPad Prism 9 software. For the open-field outcomes, we used the t-test for two related samples. A few outliers based on standard deviation were excluded before the t-test. Two-way ANOVA was used to identify possible interactions between experimental conditions and disease progression (temporal windows). To detect the potential differences between pre-infection and time windows post-instillation, we used two tailed t-tests for independent samples, or the non-parametric Mann–Whitney test where appropriate. Similarly, for the statistical analysis of the microglia data, we used two-tailed t-tests.

## Results

**Figure 2:**
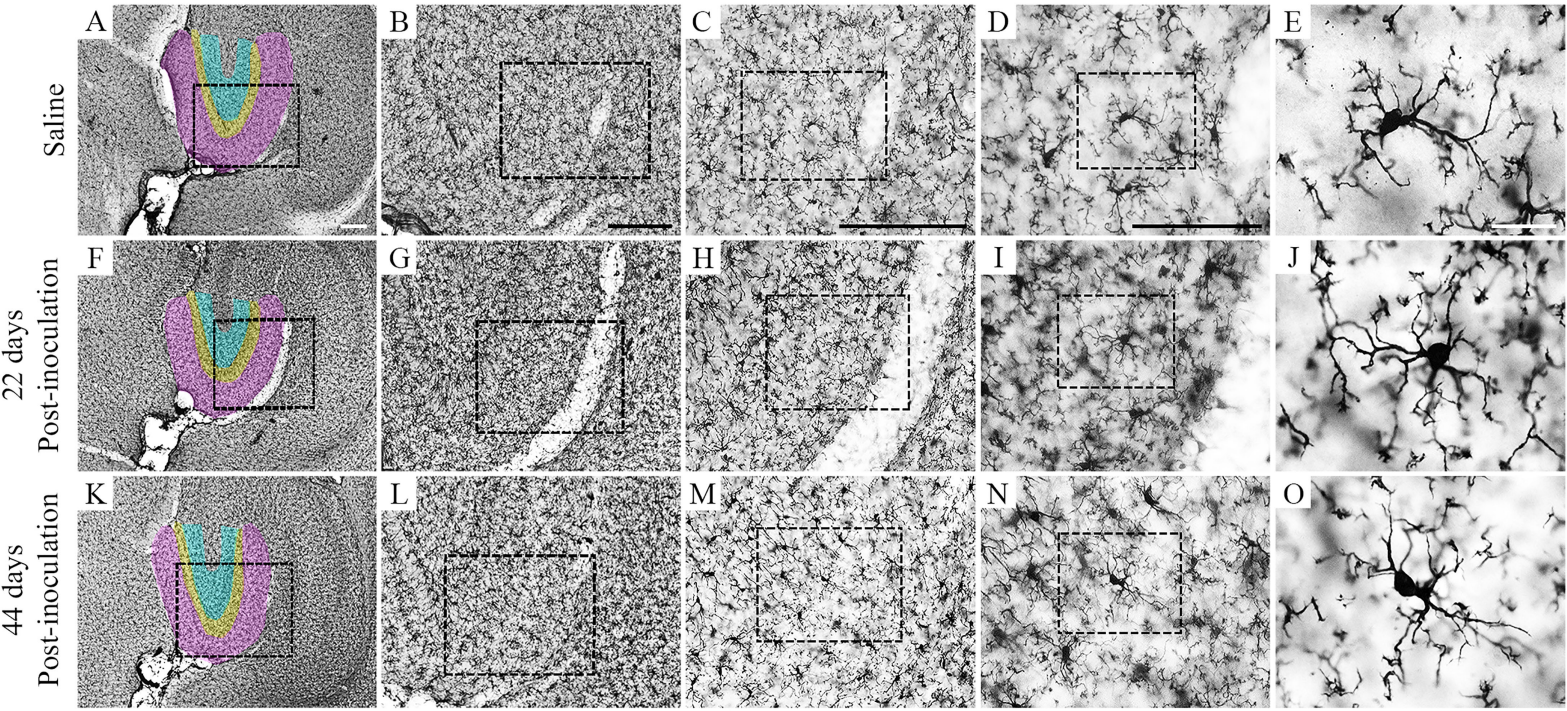
Photomicrographs of IBA1-immunolabeled brain sections from control **(A–E)** and infected mice at 22 and 44 dpi. The molecular layer of dentate gyrus is pink shaded, and granular and polymorphic layers are yellow- and blue-shaded areas in low-power picture. Dotted squares progressively show greater magnifications. Scale bars: **(A, F, K)** = 250 µm; **(B, G, L)** = 250 µm; **(C, H, M)** = 250 µm; **(D, I, N)** = 125 µm; **(E, J, O)** = 25 µm.

A random and systematic stereological sample approach was used to estimate the total number of microglia. **Figure 2** shows low- and high-power photomicrographs of brain sections indicating layers and limits of the dentate gyrus (color-shaded areas) from the control (Saline) and infected mice at 22 and 44 dpi.

**Figure 3:**
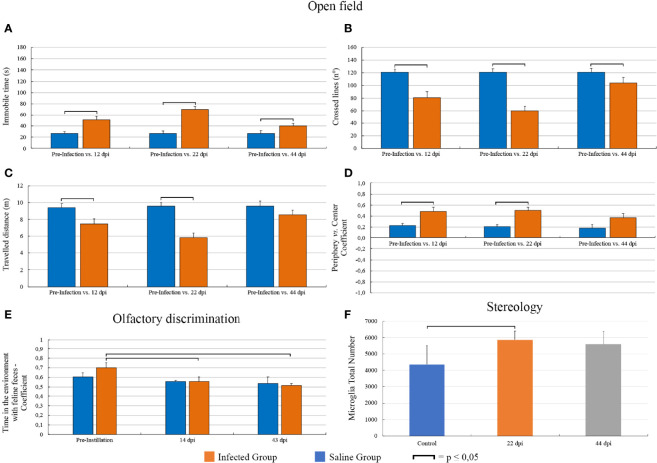
Graphic representations of *T. gondii*-induced behavioral and microglial changes as disease progressed. In the open field task, infected groups at 12, 22, and 43 dpi showed a significant increase of immobility with greater mean values at 22 dpi. In general, crossed lines and travelled distances were reduced as immobility increased. Contrast values between the times spent in the periphery and in the center of the open arena increased at 12 and 22 dpi, returning to the pre-instillation levels at 44 dpi. Infected animals increased the time spent in the feline’s odor compartment relatively to the total time test, at 14 dpi, and this altered outcome did return to baseline. The mean number of microglia at 22 dpi was greater than that of the control group. At 44 dpi, this significant difference disappeared. **(A–E)** Graphic representations display standard error bars. Microglial number graphic representation **(F)** displays standard deviation.

Compared to pre-infection levels of immobility, the infected group displayed increased immobility at 12 dpi (26.41 ± 50.6 ± 6.4, p = 0.0008, t = -4.27, n = 15), 22 dpi (26.6 ± 7.5 vs. 70.1 ± 5.4, p = 0.0001, t = -5.77, n = 14), and 43 dpi (26.45 ± 5.85 vs. 40.0 ± 4.7, p = 0.04, mean ± s.e., t = -2.31, n = 10) with greater immobility mean values at 22 dpi (**Figure 3A**). In general, line crossings (**Figure 3B**) and distance travelled (**Figure 3C**) reduced as immobility increased. Compared with pre-instillation values, contrast indices between the time spent in the periphery and in the center of the open arena also increased at 12 dpi (0.23 ± 0.04 vs. 0.48 ± 0.08, n = 17, t = -3.73, p = 0.0018) and 22 dpi (0.21 ± 0.04 vs. 0.5 ± 0.06, n = 15, t = -4.0, p = 0.0013) returning to the pre-instillation levels at 43 dpi (0.19 ± 0.06 vs. 0.37 ± 0.07, n = 10, t = -2.06, p = 0.07) (**Figure 3D**).

In the olfactory discrimination test, compared with the pre-infection behaviour, infected animals spent greater amount of time with the feline feces at 14 dpi (C_pi_ = 0.70 ± 0.05 vs. C_14dpi_ = 0.56 ± 0.05, t = 2.02, p = 0.05, n = 16) and at 43 dpi (C_pi_ = 0.73 vs. C_43dpi_ = 0.52, n = 16 vs. Z(U) = 2.14, p = 0.032, n = 8) (**Figure 3E**). No changes in the olfactory test were observed in control group after instilled saline in the ocular conjunctival sac.

The mean number of Mol-DG total number of microglia (**Figure 3F**) at 22 dpi (5,870 ± 531) was greater than that of the control group (4,349 ± 1,171), returning to the control levels at 44 dpi (5,608 ± 765; mean ± standard error). The coefficients of methodological error (Scheaffer’s coefficient of error) were smaller than 0.05, and the coefficients of biological variation were respectively 87.5%, 97%, and 94% (**Table 2**).

**Table 2:** Microglial stereological estimate in the molecular layer of dentate gyrus.

Subject	Thickness (µm)	Volume (mm³)	Number of sections	Number of boxes	Box dimensions (µm)	Grid dimensions (µm)	Dissector height (µm)	Interval between sections
					Control
09	43.4	0.7	5	492	50 × 50	70 × 70	15 µm	1/4
08	38.3	0.8	6	683	50 × 50	70 × 70	15 µm	1/4
01	24.3	0.5	5	414	50 × 50	70 × 70	15 µm	1/4
10	27.3	0.5	5	428	50 × 50	70 × 70	15 µm	1/4
22 days post-infection								
22	29.7	0.6	5	481	50 × 50	70 × 70	15 µm	1/4
26	24.9	0.7	6	585	50 × 50	70 × 70	15 µm	1/4
28	25.5	0.5	5	476	50 × 50	70 × 70	15 µm	1/4
15	28.6	0.5	5	458	50 × 50	70 × 70	15 µm	1/4
18	33.9	0.5	7	481	50 × 50	70 × 70	15 µm	1/4
44 days postinfection								
27	29.0	0.6	5	496	50 × 50	70 × 70	15 µm	1/4
24	36.6	0.6	5	528	50 × 50	70 × 70	15 µm	1/4
11	25.6	0.5	5	444	50 × 50	70 × 70	15 µm	1/4
16	30.6	0.5	5	455	50 × 50	70 × 70	15 µm	1/4
29	33.8	0.5	7	481	50 × 50	70 × 70	15 µm	1/4

Results from control and infected mice 22 days postinfection and 44 days postinfection.

**Figure 4:**
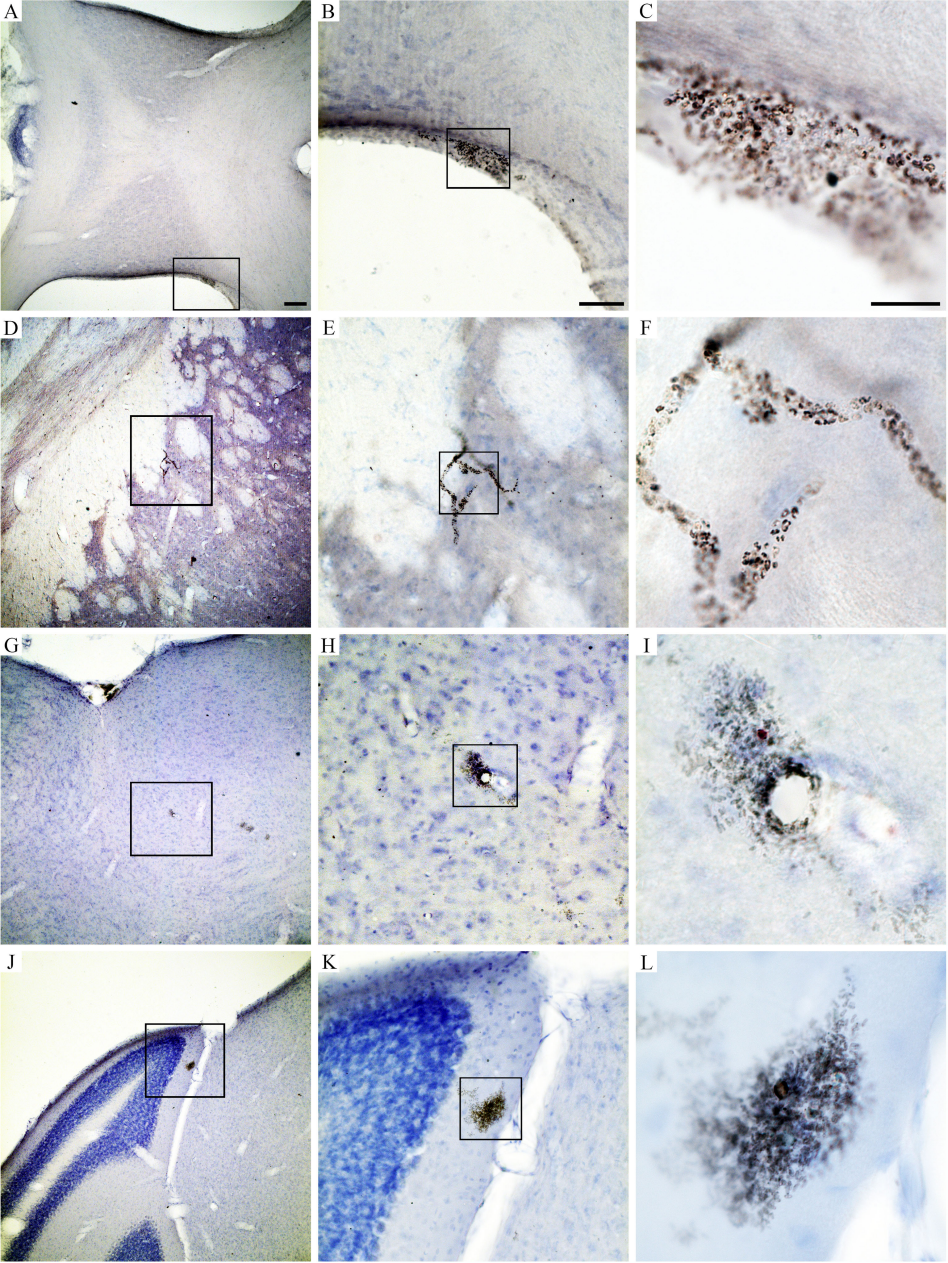
Brain sections photomicrographs of periventricular and perivascular infiltrates in the lateral septum **(A–C)**, striatum **(D–F)**, cerebral cortex **(G–I)**, and cerebellum **(J–L)** in *T. gondii*-infected animals at 22 dpi. Scale bars: **(A, D, G, J)** = 250 µm; **(B, E, H, K)** = 125 µm; **(C, F, I, L)** = 25 µm.

**Figure 5:**
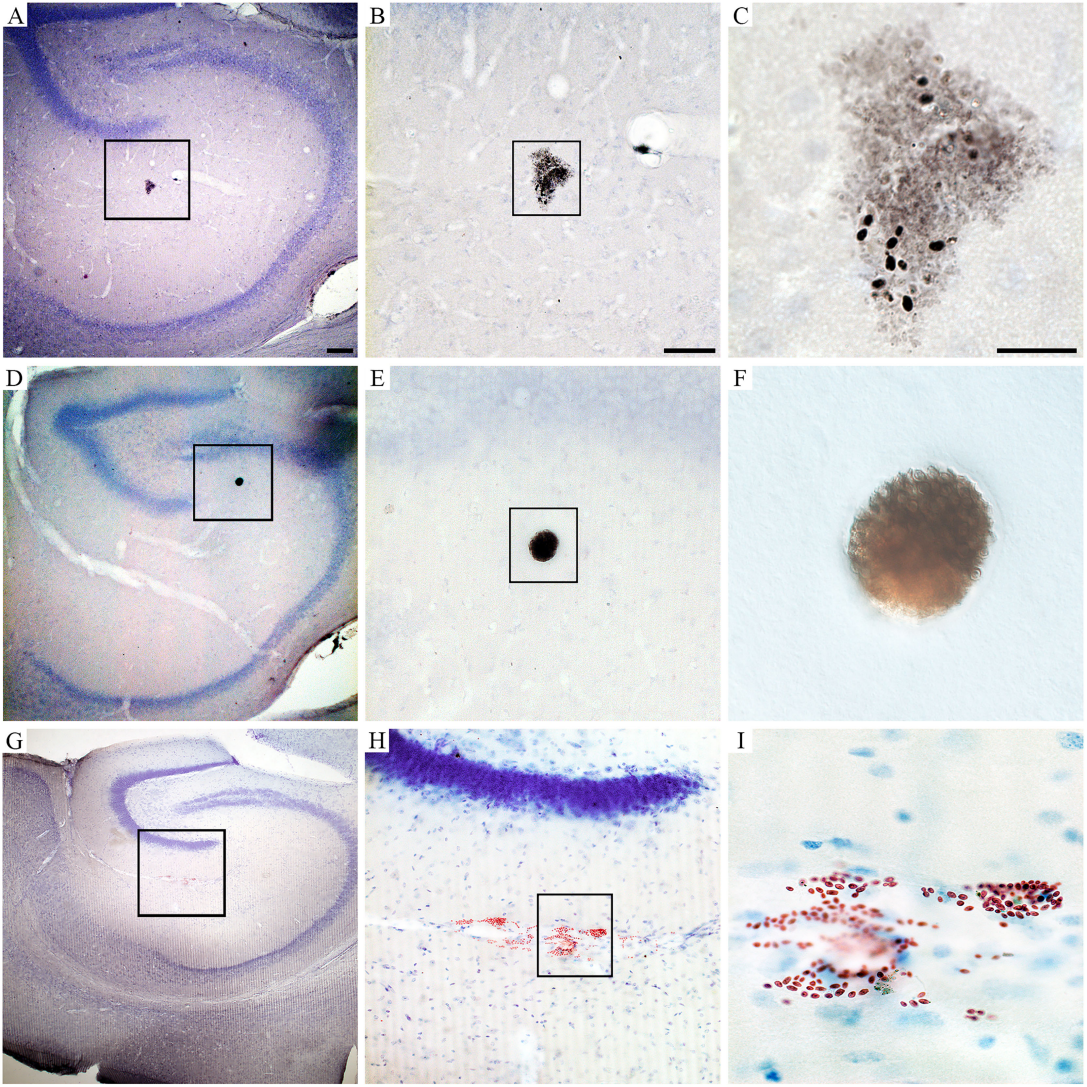
Photomicrographs of hippocampal sections to illustrate perivascular infiltrate **(A–C)**, *T. gondii* parasite encystment **(D–F)**, and vascular congestion in the hippocampus fissure **(G–I)** at 22 dpi. Scale bars: **(A, D, G)** = 200 µm; **(B, E, H)** = 100 µm; **(C, F, I)** = 30 µm.

**Figure 6:**
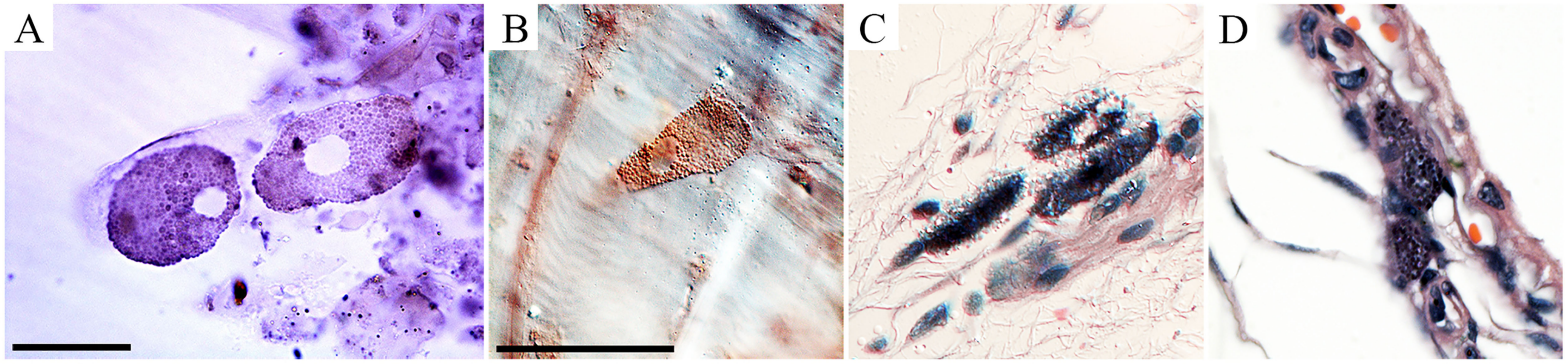
Retinal photomicrographs *T. gondii*-infected mice at 22 dpi. **(A, B)** Flat mount of retina counterstained with Giemsa after *T. gondii* immunolabeling. **(C, D)** Low- and high-power photomicrographs of retinal sections stained with hematoxylin–eosin at the same time window. **(A)** High-power pictures of two retinal *T. gondii* pseudocysts. **(B)** Interferential contrast microscopic image of a retinal *T. gondii* pseudocyst. Macrophages and polymorphonuclear cells (neutrophils) in the retinal section of infected mouse is shown in **(C, D)**. Scale bars: **(A)** = 25 µm; **(B–D)** = 50 µm.

Behavioral changes of *T. gondii*-infected mice coincided with neuropathological changes which included periventricular and perivascular infiltrates in many CNS areas, including the lateral septum, striate, cerebral cortex, cerebellum, and hippocampus (**Figures 3**, **4**) and parasite encystment in the hippocampus (**Figures 5A**–**F**). Periventricular vascular congestion was also observed in the hippocampal fissure and in the retina, (**Figures 6A–D**). Although less intense, similar neuropathological features were found at 43 dpi (not illustrated).

Three-dimensional reconstructions and correspondent dendrograms were generated for selected microglia (**Supplementary Figure 3**) from the dentate gyrus molecular layer from both infected and control mice. Compared to control, microglia from 22 and 44 dpi of infected mice showed a more ramified branching pattern, higher branch volumes, and larger trees. The dendrograms of the 3D reconstructions further confirmed the presence of increased branching of microglial processes in infected mice.

## Discussion

A previous study using C57BL/6 mice reported how the conjunctival instillation of the *T. gondii* ME-49 strain induces ocular infection, confirmed the presence of free parasites in the retinal vasculature, a mononuclear inflammatory infiltrate, and the presence of parasites in the choroid vasculature (Tedesco et al., 2005). Using ocular conjunctival instillation of *T. gondii* tachyzoites, we extended previous observations by demonstrating that the infection of BALB/c mice with *T. gondii* alters the way in which the animals explore new environments and assess risk; these changes were associated with tachyzoite differentiation into bradyzoites in-enclosed cysts within the brain and retina and with an altered microglial response in the molecular layer of the dentate gyrus.

The open field and the elevated plus maze are unconditioned tests widely used for measuring anxiety-like behavior in mice and rats. However, it has been suggested that these tests are, at best, tests of a natural preference for unlit and/or enclosed spaces (Ennaceur and Chazot, 2016). In both tasks, when animals explore an unfamiliar area, they remain close to the walls; this preference is considered to be an indication of fear-induced anxiety (Lalonde and Strazielle, 2008; Ennaceur, 2014). These tests are based on the natural tendencies in mice to avoid open and elevated areas and to spontaneously explore unfamiliar areas (Komada et al., 2008). Thus, animals appear to innately avoid open and/or lit spaces in the central area of the OF (Ennaceur, 2014). Hiding behavior may contribute to avoiding attack and predation, and it may be included in the repertoire of animal survival instincts. The species-specific hiding response is an essential component of their natural preference for unlit and protected spaces. Thus, mouse preference for the safety of the peripheral zone of the OF may reflect this adaptive response; for a recent review, see (Ennaceur, 2014).

Horizontal locomotor exploration is a component of the innate repertoire of benaviours used by animals to explore novel environments and to assess risk in the wild (Augustsson and Meyerson, 2004; Ennaceur, 2014). It has been suggested that this strategy arises from the drive to avoid and to explore a perceived threatening stimulus (Crusio, 2001). In the present study, this stereotypical behavior was clear when control and infected mice explored the open arena (Lister, 1990; Kalueff et al., 2006; Ennaceur, 2014). However, the way that infected mice explored the open arena, compared to the controls in the same time periods, changed considerably after infection, and this was evidenced by increased immobility and an increased contrast ratio: more time was spent in the periphery than in the center at 12 and 22 dpi. These results may reflect either an increased risk assessment or a decrease in exploratory activity associated with the development of sickness behaviors (Dantzer et al., 2008), a strategy for adaptive energy reallocation (Pacheco-López and Bermúdez-Rattoni, 2011). Because the olfactory discrimination test suggested that infected mice spent a greater amount of time in the feline’s odor compartment, we reasoned that increased risk assessment for predation unlikely to account for the open-field results. In addition, at 43 dpi, the return to pre-instillation levels, for time spent in the central and peripheral regions of the of also suggest that the greater immobility in infected mice is not due to increased risk assessment. Thus, we suggest that the greater immobility of infected mice may be a consequence of sickness behavior owing to energy reallocation during the acute phase of *T. gondii* infection (Pacheco-López and Bermúdez-Rattoni, 2011), whereas the olfactory test results in the infected mice suggested a reduction of predator avoidance behavior. However, the dentate gyrus is involved in memory dysfunction, and the question of whether the resulting microglial response weakens learning or reduces fear in mice needs to be considered. Indeed, previous findings indicated that T. gondii infection in a C57Bl6 mouse model caused anxiety-like behavior (increasing time spent in the corners as compared to the center of the open field), alteration in social novelty, and severe impairment of spatial memory, and this was associated with significant loss of NMDAR expression and signs of neurotoxicity, neurodegeneration, and cell death, resulting in cognitive impairment (Torres et al., 2018). T. gondii infection induces neutrophil, microglial, and dendritic cell recruitment to the site of the infection which may lead to the release of cytokines (Araujo and Slifer, 2003; El Saftawy et al., 2021) that are necessary to promote the killing of the parasite and inhibit its replication (Dupont et al., 2012). In the present report, we found a significant increase of microglia and macrophage infiltrates the molecular layer of the dentate gyrus, a target layer of the entorhinal cortical projections associated with object recognition and spatial memories (van Groen et al., 2003) which may be impaired in the BALB/c microglial response in the dentate gyrus of infected mice. It is important to highlight that although the association between the increase in number of microglia in the molecular layer of the dentate gyrus of infected mice, and the behavioral changes seem to be correlated. The argument would be different if another higher order brain area, less involved in learning and memory also exhibited similar association, and this is an important limitation of the present study. Indeed this is an important limitation of the present study that could have been avoided if another area was explored. Similarly, it has been previously suggested that T. gondii infections in mice exhibit a consistent pattern of monocyte infiltration 14 days after T. gondii infection to the olfactory tubercle, an area involved in olfactory processing and perception (Wesson and Wilson, 2011). Thus, it may be possible that olfactory memory dysfunction may occur in T. gondii-infected mice, and this may be associated with this selective neuroinvasion (Schneider et al., 2019) and/or with the impairment of olfactory and fear memory areas such as the olfactory tubercle, olfactory cortex, and amygdala, which are involved in behavioral decisions (Mori and Sakano, 2021).

Although it has been suggested that adult-born neurons are engaged in the retrieval of olfactory associative memory in the dentate gyrus (Kedrov et al., 2019), and in the present report we found significant changes in microglial in this area, in association with altered olfactory tests in infected mice, these findings remain to be explored in detail in future investigations.

In the present report, behavioral changes were associated with neuropathological changes in *T. gondii-*infected mice and this included tachyzoites to bradyzoite encystment, a microglial response, and inflammatory infiltrations in many CNS areas.

Microglial proliferation has been associated with chronic *T. gondii* infection, and in the brain, the parasite induces the expression of anti-inflammatory cytokines and contributes to inhibiting toxoplasmic encephalitis through suppression of cytokine signaling 1 and Arg1 (Hwang et al., 2018). Transcriptome studies have indicated that homeostatic microglia gradually adopt a unique phagocytic disease-associated microglia (DAM) phenotype in neurodegenerative disease, chronic inflammation, and advanced aging (Rangaraju et al., 2018). In addition, it has been demonstrated that *T. gondii* infection induces homeostatic microglial proliferation that reduces Aβ plaque burden in a 5XFAD AD mouse model (Shin et al., 2021), suggesting a sustained supply of homeostatic microglia may help in the resolution of Alzheimer’s and other prion-like neurodegenerative disorders.

In the present report, we found evidence for microglial proliferation at 22 dpi in *T. gondii*-infected mice followed by a reduction to control levels at 43 dpi. Because microglia and macrophages are the major producers of interferon-gamma in the brain following infection with *T. gondii* (Suzuki et al., 2005) and interferon-gamma is the major effector molecule controlling tachyzoite behavior through the expression of chemokines and MHC antigens (Wang et al., 2004; Wang and Suzuki, 2007; Sa et al., 2015), we suggest that increased microglia in CNS-infected areas may have prevented toxoplasmic encephalitis in BALB/c-infected individuals, limiting parasite proliferation at the early stage of the disease. It is important to note that IFN-γ, which is considered to be produced exclusively by lymphoid cells, has been shown to be produced by murine microglia in response to IL-12 and/or IL-18 (Kawanokuchi et al., 2006).

Encystment requires the expression of a new repertoire of surface antigens by the parasite inside a modified parasitophorous vacuole, during the early stage of systemic infection (Radke et al., 2018; Bergersen et al., 2021). At 28 dpi, the pattern of parasite-specific gene expression in the C57BL/6 mouse suggested the establishment of a chronic infection dominated by a late-stage bradyzoite phenotype, and this parasite stage-specific gene expression correlates with host gene changes (Carrera et al., 1996; Bergersen et al., 2021).

The kinetics of the host response to chronic infection and the regulation of the neuroinflammatory response are critical contributors to disease outcome. An efficient host immune response, exhibiting a classical immune cell activation and increased activation of IFNγ signaling-related genes, is essential to avoid *Toxoplasmic encephalitis* (Shen et al., 2001). All T-cells and CD8+ T cells more specifically (Landrith et al., 2015; Khan et al., 2019), increase in number in infected mice, and this is associated with the increased number of macrophages and activated microglia until 28 dpi, when they stabilize (Bergersen et al., 2021). However, parasite virulence and persistence in mice are strongly dependent on the host genetic background (Behnke et al., 2016). Here we investigated neuropathological changes at 22 and 43 dpi in BALB/c mice, which has been described as being less susceptible to *T. gondii* infection (Mukhopadhyay et al., 2020). Indeed, it has been shown that at each stage of infection, lesions in BALB/c mice exhibit altered cellular responses compared with those in the C57BL/6 mouse model; there appear to be an increased number of macrophages in the late stages (56 dpi) of disease in the BALB/c mice, but the total number of infiltrating leukocytes seems to be downregulated at 28 and 56 dpi in the BALB/c mice. These events were associated with less immune cell recruitment and better control of the parasite. Concurrently, the activation pathways associated with neuropathology and neuroinflammation in BALB/c mice were downregulated compared with corresponding pathways in C57BL/6 (Bergersen et al., 2021). Thus, gene expression, immune cell recruitment, and activation of neuroinflammatory pathways seem to be more efficient in BALB/c, and this is associated with less marked neuropathology and faster resolution in the *T. gondii*-infected BALB/c model. Our findings related to systemic behavioral changes reaching a peak at 22 dpi followed by a reduction at 43 dpi, associated with an increase followed by a decrease of microglia, the most important component of the cellular innate immune response at the same time windows, seem to reflect the events at the molecular level. Thus, the sickness behaviors we observed are more consistent with parasite clearance and a return to homeostasis rather than the expression of the previously reported increases in risk-taking behaviors in other strains that would be more consistent with chronic infection and encystment of the parasite in the brain. It remains unclear what is the basis for these strain differences, but a better understanding of the mechanisms underpinning the host response in different mouse strains may lead to the development of therapies that might help to overcome the dangers of neurotoxoplasmosis.

The present study demonstrated that behavioral changes in rodents infected with T. gondii are influenced by the genotype of the infecting strain (Bezerra et al., 2019). In BALB/c mice, the influences of genetic background were tested using ME-49 (type II) and VEG (type III) strains, and humoral immune response and the number of parasites in the CNS were assessed after behavioral tests for learning and memory, locomotor activity, and aversion of feline odor. The authors found that VEG background showed greater levels of total IgG anti-toxoplasma, higher tissue burden of T. gondii in the CNS, long-term memory impairment, lower mobility, and lower aversion of feline odor than mice infected with the ME-49 strain (Bezerra et al., 2019). With these results in mind, we decided to test the VEG strain using the less invasive pathway of ocular instillation. We found similar neuropathological features and behavioral changes as described elsewhere and confirmed that ocular instillation is an effective pathway to induce experimental toxoplasmosis infection. To further explore the pathway of experimental toxoplasmosis infection in detail, future studies might integrate behavioral, cellular, and molecular approaches using other parasite genetic backgrounds, different brain areas, and distinct mouse models (Desmettre, 2020).

## Data Availability Statement

The original contributions presented in the study are included in the article/**Supplementary Material**. Further inquiries can be directed to the corresponding author.

## Ethics Statement

The experiments were conducted in accordance with the recommendations in the Guide of the National Institutes of Health (NIH, USA), for the use of experimental animals and in accordance with the ethics committee of the Institute of Biological Sciences at the Federal University of Para – UFPA, under the Protocol No. 7961160818 and Protocol No. 11/2013 of Evandro Chagas Institute. In this study, the parasite sample of *T. gondii* used was obtained from the Laboratory of Toxoplasmosis of the Evandro Chagas Institute - IEC. All efforts were made to minimize the number of animals used and the stress and discomfort to animals.

## Author Contributions

All listed authors contributed substantially to the conception or design of the work; the acquisition, analysis, or interpretation of data for the work; drafting the work or revising it critically for important intellectual content; and/or final approval of the version to be published; and agreed to be accountable for all aspects of the work in ensuring that questions related to the accuracy or integrity of any part of the work are appropriately investigated and resolved. CD, DD, JD, and DA participated in the data interpretation and writing of the final version.

## Funding

CD was supported by the Brazilian Research Council – CNPq Grant No. 307749/2004-5 and 471077/2007-0, Fundação Amazônia de Amparo a Estudos e Pesquisas do Pará – FAPESPA, ICAAF No. 039/2017, Pró-Reitoria de Pesquisa e Pós-Graduação da Universidade Federal do Pará – PROPESP Edital 2021-PIAPA; Coordenação de Aperfeiçoamento de Pessoal de Nível Superior – CAPES – Pró-Amazônia, Grant No. 3311/2013; and Programa de Apoio à Publicação Qualificada – PAPQ/PROPESP/UFPA.

## Conflict of Interest

The authors declare that the research was conducted in the absence of any commercial or financial relationships that could be construed as a potential conflict of interest.

## Publisher’s Note

All claims expressed in this article are solely those of the authors and do not necessarily represent those of their affiliated organizations, or those of the publisher, the editors and the reviewers. Any product that may be evaluated in this article, or claim that may be made by its manufacturer, is not guaranteed or endorsed by the publisher.

## Supplementary Material

Supplementary Figure 1 Schematic diagrams of the open field **(A)** and olfactory discrimination **(B)** apparatus. The OF apparatus consisted of a grey polyvinyl chloride box (30 x 30 x 40cm). The floor was digitally divided into central and peripheral regions of equal areas by Anymaze software, and the time spent in each digital compartment was recorded. Olfactory discrimination apparatus had three equal size compartments (16 x 27 x 12cm) placed side by side and connected with polyvinyl tubes allowing mice to change between compartments. The time spent in each compartment was recorded.

Supplementary Figure 2 Stereological sampling approach to estimate the total number of microglia in the molecular layer of dentate gyrus. The pink area defines the boundaries of the molecular layer of the dentate gyrus **(A)**. Systematic random placement of the counting boxes are over the area of interest as defined by the red outline **(B)**. Cell bodies inside the box or over the box green borders were included in the count while those crossing the red borders were excluded. Scale bars: A =250µm; B =140µm.

Supplementary Figure 3 Three-dimensional reconstructions of selected microglia from the molecular layer of the dentate gyrus. Left, middle, and right columns correspond to representative microglia from brains sections of control mice, 22dpi, and 44dpi, respectively. Individual branches were distinctively colored to facilitate examination. Linear dendrograms of microglial arbors are shown below each 3D reconstruction. The length of each branch segment is displayed to scale; sister branches are horizontally displaced. Branch colors correspond to the 3D reconstructions above. Dendrograms were plotted and analysed with Neuroexplorer (MicroBrightField). Scale bars: 25µm.
