# Supplementary figures and images for: Behavioral and Neuropathological Changes After Toxoplasma gondii Ocular Conjunctival Infection in BALB/c Mice
Source: Front Cell Infect Microbiol. 2022 Mar 9;12:812152. doi: 10.3389/fcimb.2022.812152 (PMC8965508; doi:10.3389/fcimb.2022.812152)

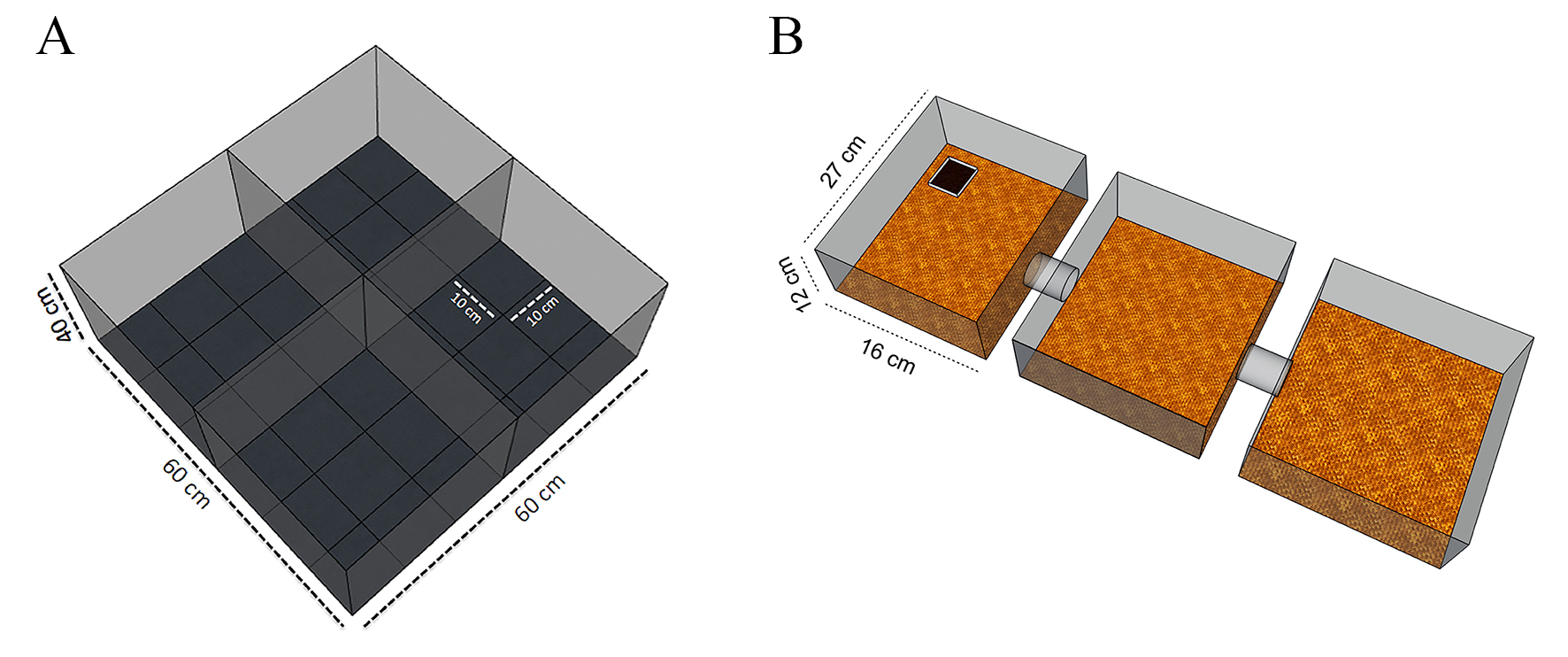

Supplement: Supplementary Figure 1 — Schematic diagrams of the open field (A) and olfactory discrimination (B) apparatus. The OF apparatus consisted of a grey polyvinyl chloride box (30 x 30 x 40cm). The floor was digitally divided into central and peripheral regions of equal areas by Anymaze software, and the time spent in each digital compartment was recorded. Olfactory discrimination apparatus had three equal size compartments (16 x 27 x 12cm) placed side by side and connected with polyvinyl tubes allowing mice to change between compartments. The time spent in each compartment was recorded. [file Image_1.jpeg]

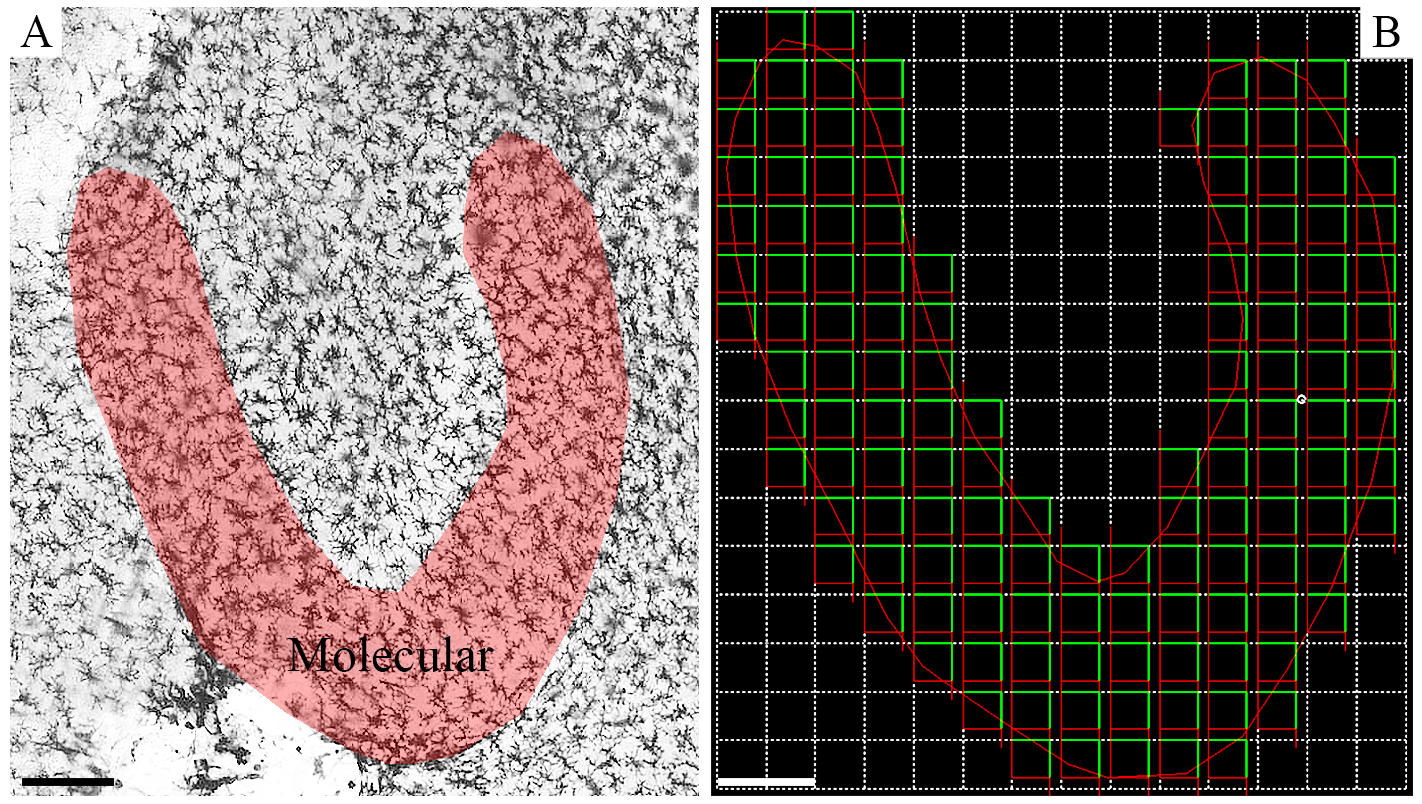

Supplement: Supplementary Figure 2 — Stereological sampling approach to estimate the total number of microglia in the molecular layer of dentate gyrus. The pink area defines the boundaries of the molecular layer of the dentate gyrus (A). Systematic random placement of the counting boxes are over the area of interest as defined by the red outline (B). Cell bodies inside the box or over the box green borders were included in the count while those crossing the red borders were excluded. Scale bars: A =250µm; B =140µm. [file Image_2.jpeg]

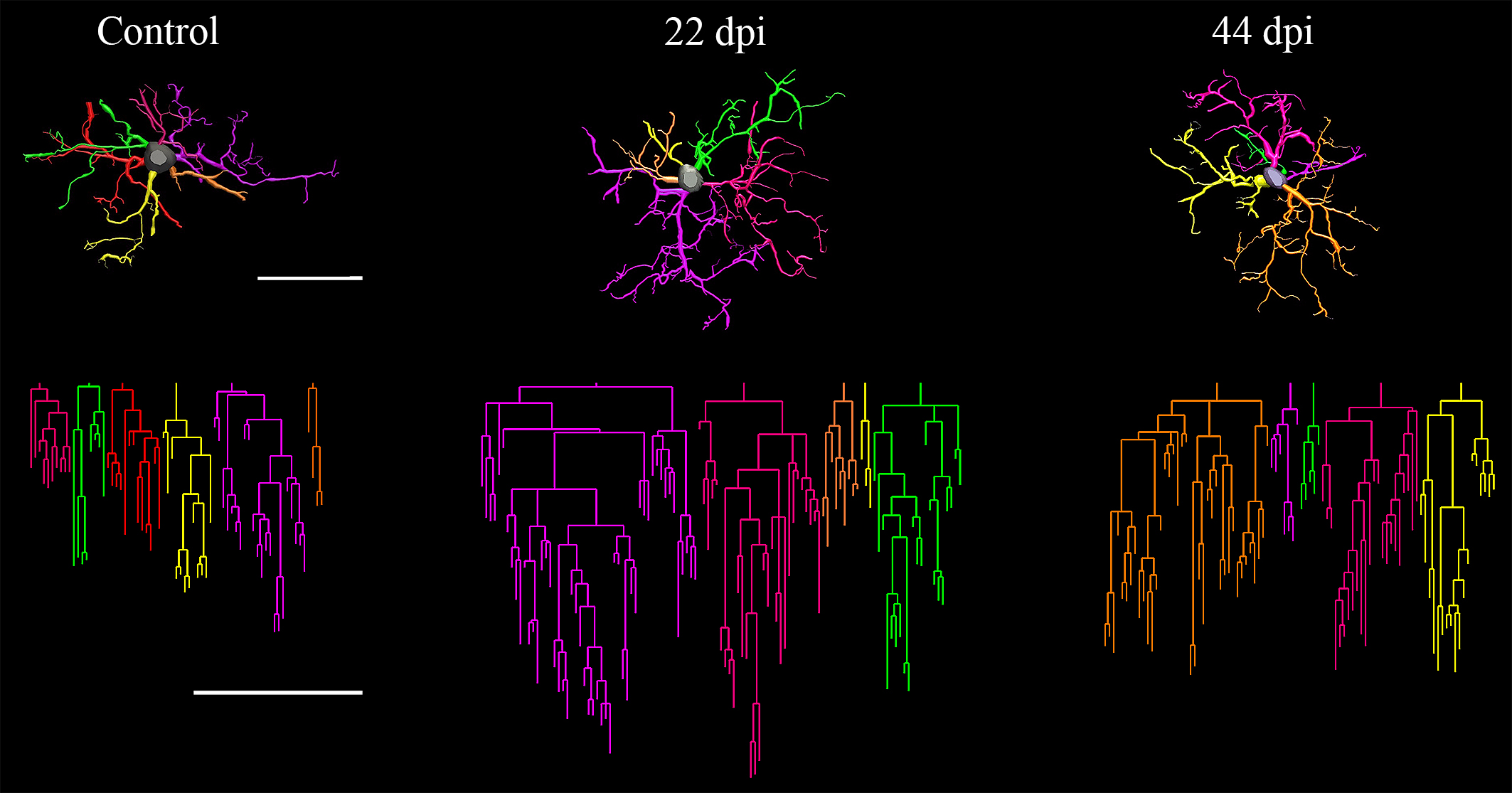

Supplement: Supplementary Figure 3 — Three-dimensional reconstructions of selected microglia from the molecular layer of the dentate gyrus. Left, middle, and right columns correspond to representative microglia from brains sections of control mice, 22dpi, and 44dpi, respectively. Individual branches were distinctively colored to facilitate examination. Linear dendrograms of microglial arbors are shown below each 3D reconstruction. The length of each branch segment is displayed to scale; sister branches are horizontally displaced. Branch colors correspond to the 3D reconstructions above. Dendrograms were plotted and analysed with Neuroexplorer (MicroBrightField). Scale bars: 25µm. [file Image_3.jpeg]
